# Distribution of inorganic compositions of Japanese tap water: a nationwide survey in 2019–2024

**DOI:** 10.1038/s41598-024-65013-4

**Published:** 2024-06-19

**Authors:** Mayumi Hori, Katsumi Shozugawa, Tsutomu Takizawa, Yuichiro Watanabe

**Affiliations:** 1 Komaba Organization for Educational Excellence, The University of Tokyo, 3-8-1 Komaba, Meguro, Tokyo, 153-8902 Japan; 2 Graduate School of Arts and Sciences, The University of Tokyo, 3-8-1 Komaba, Meguro, Tokyo, 153-8902 Japan

**Keywords:** Tap water, Quality assessment, Inorganic components, Distribution, Japan, Environmental chemistry, Environmental monitoring

## Abstract

A nationwide survey of inorganic components of tap water all over Japan was conducted from 2019 to 2024. In this survey, 1564 tap water samples were collected, and an additional 194 tap water samples were collected from 33 other countries. The water samples were analyzed for 27 dissolved inorganic components, with a primary focus on the distribution of major and trace components, including Ca, Mg, K, Na, Cl^−^, NO_3_^−^, SO_4_^2−^, total-hardness, Al, Fe, Cu, Mn, and Zn. The Japanese tap water hardness was 50.5 ± 30.2 (± 1*σ* SD) mg/L, classified as soft water according to the World Health Organization (WHO) classification. The average content of each major component in Japanese tap water tended to be lower than those in other countries. Furthermore, Piper trilinear diagrams were used to categorize Japanese tap water types. The dominating water types were the Ca–HCO_3_ and mixed types, which had a nationwide distribution. Japanese tap water generally complied with Japanese and WHO drinking water criteria, with only 1% (17/1564 sites) of the samples exceeding water quality standards. Observations of water quality changes for 2 years at three household faucets revealed that fluctuations in major components and trace metals (Al, Fe, Cu, Mn, and Zn) varied in different patterns. This suggests that the behavior of trace metal elements is influenced by local infrastructure, such as supply pipes, distinct from the variability in source water quality.

## Introduction

Water is a resource necessary for all aspects of life. It is complexly linked to various socioeconomic aspects, including human health, food production, energy production, and climate change. Thus, investigating the quality of drinking tap water is important not only for evaluating the presence of contaminants but also for risk management, public health, economic growth, and decision-making at household and community scales. The regional overview of the chemical composition of tap water is potentially crucial for water utility providers, industries planning water usage, public health authorities, researchers, and water consumers^1^. Therefore, investigating the current water quality is important to provide essential tools (fundamental dataset) and a platform for understanding various components impacting the water environment. However, although comprehensive and overview research covering major and trace components in drinking tap water has been conducted, especially in European countries^2–7^, no report exists on a nationwide survey of drinking tap water quality in Japan.

In Japan, the water supply coverage ratio is over 98%, providing a stable supply of safe drinking water^8,9^. Water quality is managed by local governments. The source of tap water also differs depending on the municipality. Tap water is derived from various sources, including surface (river) water, groundwater, spring water, lake water, and artificial reservoirs. Japanese tap water sources have a higher proportion of surface water than groundwater, with 80% surface water and 20% groundwater^10^. Although each municipality monitors water quality, the datasets used are not uniform and access to available data is limited^11^. Particularly, nonhazardous substances are rarely monitored, and the corresponding data are rarely reported. In addition, water quality monitoring covering normative items in each municipality is mainly conducted at designated points, such as the water purification plant, water pipes, and faucets. Domestic plumbing and private-supply tap water are rarely monitored as these are uncontrolled. As such, there is a significant lack of data on water quality in private settings, such as homes, where consumers utilize tap water. From the consumer perspective, accessing a centralized source of information on general water quality parameters, not just trace components, is difficult. Thus, water quality cannot be easily compared with those of other regions. Consequently, consumers are dissatisfied with the safety of tap water, and the consumption of bottled water is increasing year by year.

Japan is currently in the process of replacing water pipes; the replacement is being done sequentially. The increasing occurrence of natural disasters, such as torrential rains and earthquakes, is also a concern in worsening water quality due to the damage and deterioration of old water pipes. The continued use of deteriorated water pipes may change water quality because of its breakage and corrosion. For instance, the Great East Japan Earthquake and tsunami, which occurred in March 2011, caused catastrophic damage to the water supply infrastructure, resulting in widespread water pipe breaks. The water supply of approximately 2.57 million households was cut off, and the longest water outage was 5 months^12^. Meanwhile, the Noto Peninsula Earthquake hit on January 1, 2024, and the number of broken water pipes per kilometer was 2.66 in the hardest-hit town, approximately seven times higher than that of the Great East Japan Earthquake^13^.

In 2011, the earthquake-resistance rate of the main water pipes (water supply pipes and main distribution pipes) was 30.3% on a national average. Even 10 years later in 2022, it still stood at only 41.2%^14^. Because of the lack of funds and manpower, the replacement and earthquake-proofing of water pipes have not kept pace with current situations. In addition, the privatization of water supply services has been promoted, potentially leading to the worsening of water quality management and nondisclosure of water quality information. Therefore, continuous monitoring is required to sustain current quality.

Thus, we have conducted a comprehensive analysis using a uniform methodology at a single laboratory to evaluate the tap water quality throughout Japan since 2019. The main objective of this study is to investigate the distribution of various inorganic chemical components covering major and trace components and characterize Japanese tap water quality. We already reported the distribution of hardness in Japanese tap water for 665 sites in 2021^15^, and we aim to continuously collect tap water and reach 1564 sampling sites across Japan by 2024. We also collected tap water samples from 33 countries and compared them with Japanese tap water to classify the Japanese water quality. In our study, 27 inorganic components, 22 elements for inductively coupled plasma optical emission spectroscopy (ICP-OES), and five anions for using ion chromatography (IC) were analyzed to investigate the distribution and characteristics of water quality. Here, we report a nationwide survey of inorganic components in water and their distribution patterns across Japan. In addition, to clarify the changes in water quality in detail, we observed annual variations in major components and selected trace metal elements for 2 years. Furthermore, these results were compared with a normative set by Japanese regulations and World Health Organization (WHO) guideline values to evaluate its quality. This study provides a basic dataset for tap water quality in Japan, especially its major and trace inorganic components.

## Results and discussion

### Distribution of major inorganic components

**Figure 1:**
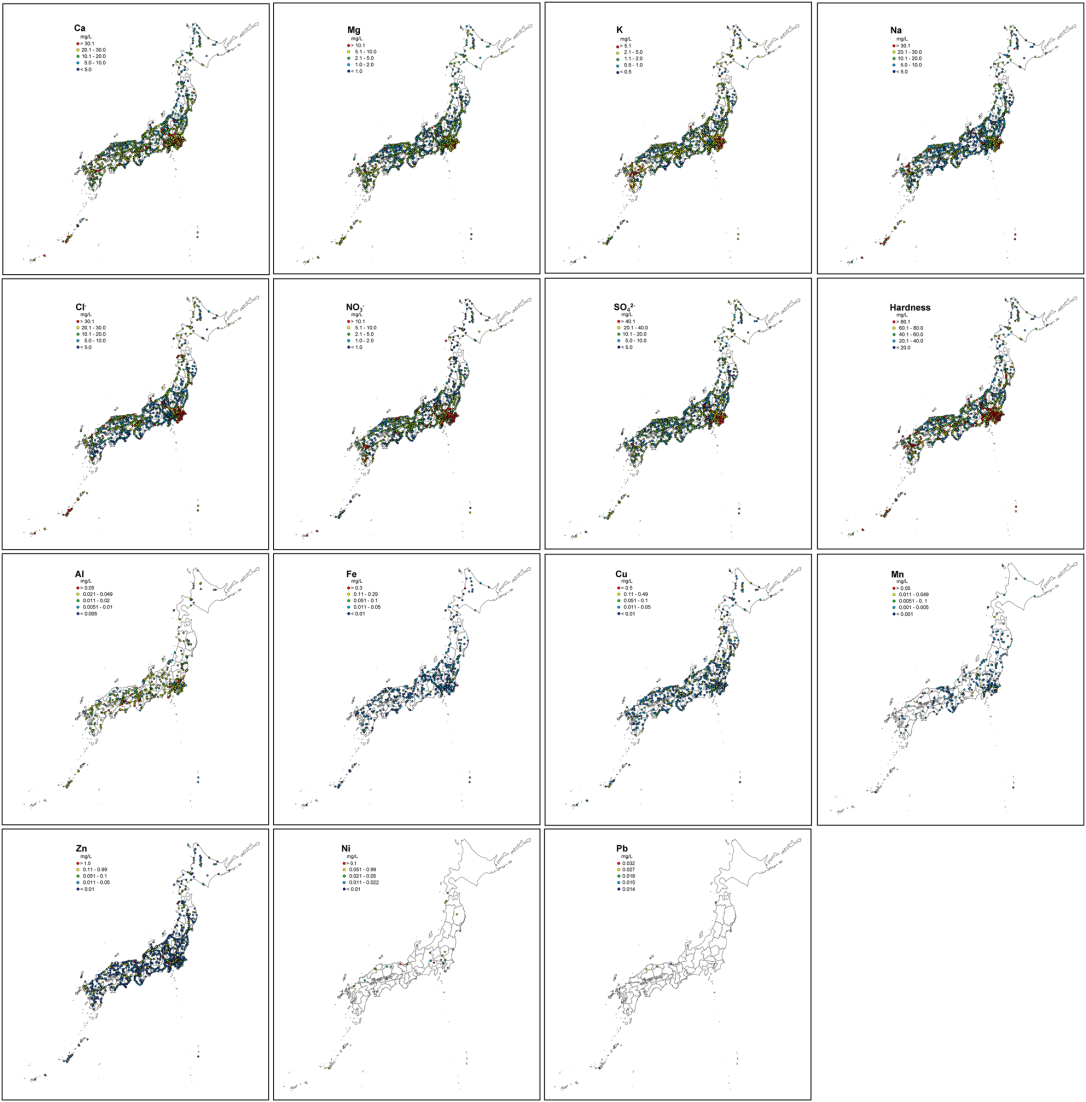
Maps of Japan showing the distribution of major and trace components in tap water (*n* = 1564). The maps were generated using ArcGIS Pro, version 2.9.3 (ESRI).

Tap water samples were collected from 1564 sites across all of Japan and from 194 sites in 33 other countries between 2019 and 2024. Of the 27 inorganic components measured, 20 components (Al, Ba, Ca, Cd, Cr, Cu, Fe, K, Mg, Mn, Na, Ni, Pb, Sr, Zn, Br^−^, Cl^−^, NO_3_^−^, SO_4_^2−^, and PO_4_^3−^) were detected in tap water samples. The distribution maps for major and trace components across Japan are shown in Fig. 1. The analytes in Japanese tap water are summarized in Table 1. Each component, both major components and trace metal elements (e.g., Zn, Mn, Cu, and Fe), exhibited wide ranges of concentrations with a broad distribution of 2–3 orders of magnitude.

**Table 1:** Analytes components in Japanese tap water, Japanese normative standard values^34,35^, and WHO guideline values^22^.

Component	*n*^a^	Minimum (mg/L)	Median (mg/L)	Maximum (mg/L)	Average (mg/L)	SD (mg/L)	Japanese drinking water quality standards^a^ ^34^ (mg/L)	WHO guidelines^a^ ^22^ (mg/L)
Ca	1564	0.04	13.8	114	14.6	8.3	–	–
K	1564	0.01	1.3	26.7	1.7	1.6	–	–
Mg	1560	0.08	2.6	64.2	3.4	3.1	–	–
Na	1564	0.52	8.2	241	10.6	10.6	200 (1)	200 (1)
Hardness	1564	0.10	46.0	511	50.5	30.2	300 (1)	–
Cl^−^	1563	0.82	8.9	719	13.6	24.4	200 (5)	250 (5)
NO_3_^−^	1523	0.01	2.2	56.0	3.6	4.7	44.3^b^ (1)	50 (1)
SO_4_^2−^	1562	0.15	9.6	217	13.9	13.4	–	500
Al	670	0.001	0.014	0.099	0.019	0.013	0.2	0.2
Ba	1517	0.0001	0.006	0.265	0.009	0.013	–	0.7
Be	0						–	–
Bi	0						–	–
Cd	1			0.003			0.003 (1)	0.003 (1)
Co	0						–	–
Cr	2	0.003	0.004	0.005	0.004	0.001	0.05^c^	0.05
Cu	849	0.001	0.014	0.981	0.041	0.100	1	2
Fe	567	0.003	0.008	0.431	0.014	0.024	0.3 (1)	–
Ga	0						–	–
Li	0						–	–
Mn	311	0.0004	0.001	0.153	0.004	0.016	0.05 (4)	0.4
Ni	28	0.007	0.025	0.265	0.043	0.052	0.02^d^ (21)	0.07 (4)
Pb	5	0.014	0.018	0.032	0.021	0.007	0.01 (5)	0.01 (5)
Se	0						0.01	0.01
Sr	1564	0.001	0.050	0.533	0.061	0.046	–	–
Tl	0						–	–
Zn	1324	0.0004	0.007	3.233	0.038	0.166	1 (8)	3 (1)
Br^−^	1444	0.01	0.05	17.6	0.08	0.47	–	–
PO_4_^3−^	7	0.392	0.8	4.6	1.5	1.4	–	–

The number of samples exceeding the standard/guideline is indicated in parentheses.

*n*: number of detected samples; SD: standard deviation (1σ).

^a^Standard/guideline value (number of exceeding samples).

^b^10 mg/L as nitrate–nitrogen.

^c^Hexavalent chromium.

^d^Guideline value (complementary item)^35^.

**Figure 2:**
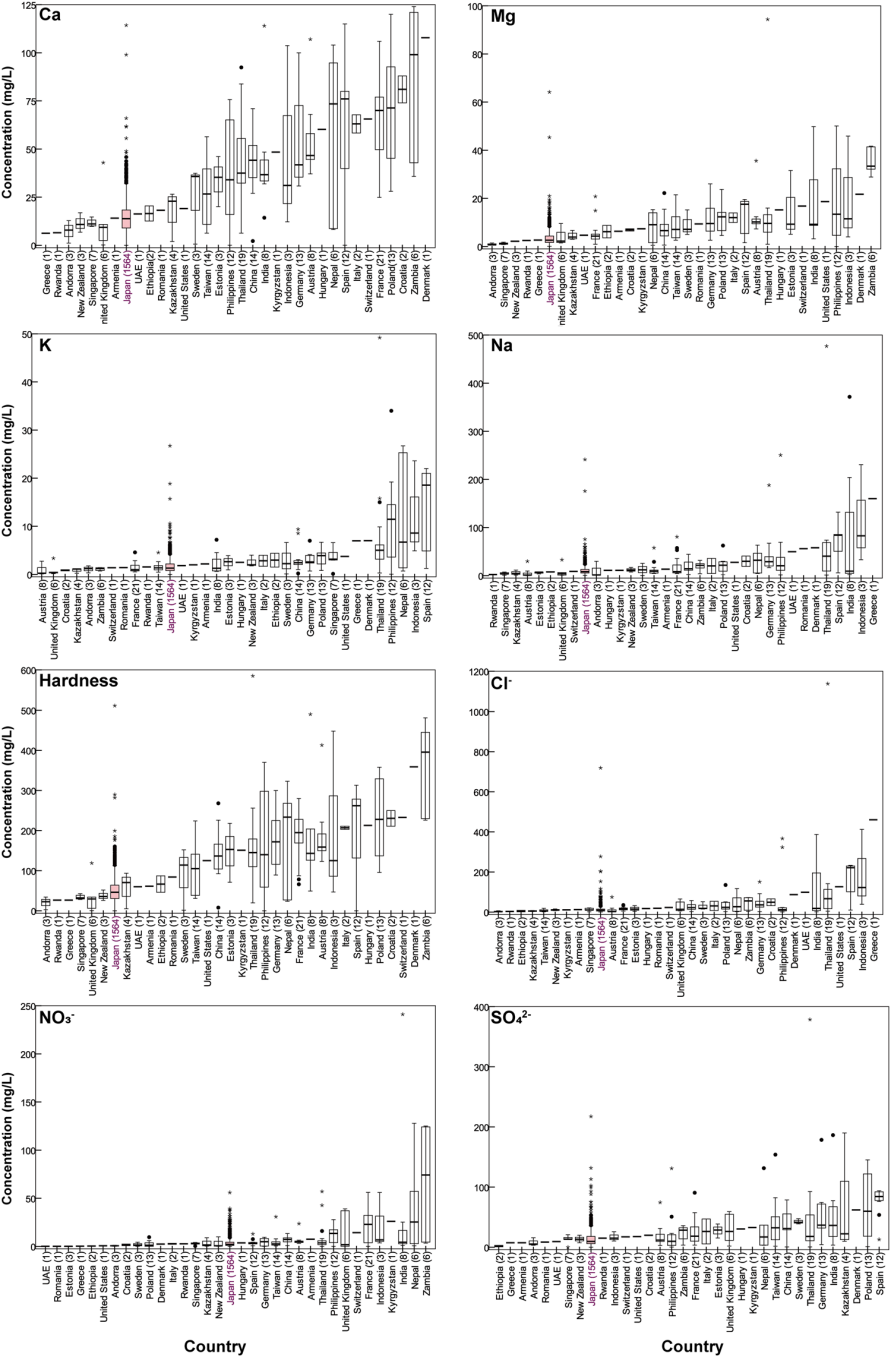
Boxplots showing the distributions of major components in tap water by increasing the national average. Boxes show the interquartile range, with a thick line at the median. Whiskers represent the nonoutlying extraquartile range. Outliers are shown as circles (near outliers) or asterisks (far outliers).

**Figure 3:**
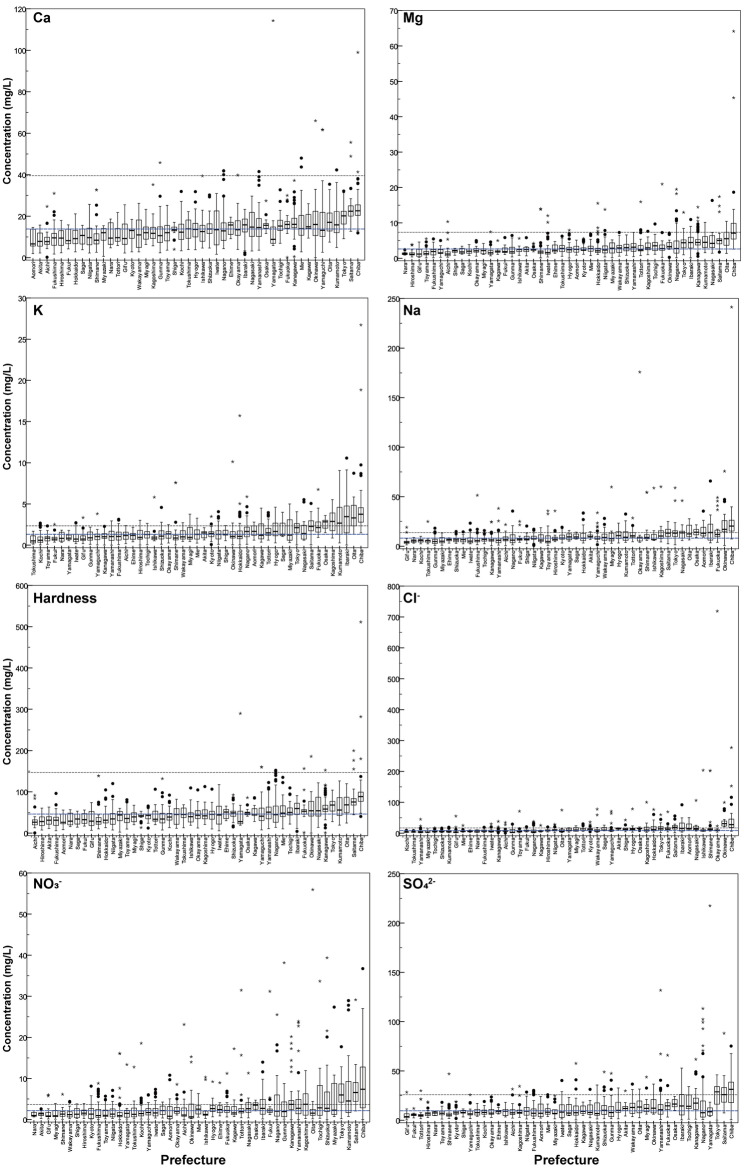
Distribution of major components in Japanese tap water based on increasing the prefecture average (for the prefecture location, see Fig. S1). Boxes show the interquartile range, with thick lines at median values. Whiskers represent the nonoutlying extraquartile range. Outliers are shown as circles (near outliers) or asterisks (far outliers). Black dashed lines indicate the median of tap water collected worldwide (*n* = 194). Dashed blue lines indicate the median of Japanese tap water samples (*n* = 1564).

The tap water quality was found to be widely distributed throughout Japan. Compared with other published data covering the same components in tap water from Italy (*n* = 157) and European countries (*n* = 579)^3,4^, our findings similarly showed wide ranges of concentrations spanning several orders of magnitude for many components. Figures 2 and 3 show a boxplot of the distribution of major components for the collected tap water by country and Japanese prefectural averages, respectively. Therein, there are wide variations in the components of tap water not only between countries but also within the same country.

Among major cations, the average concentrations of Ca, Mg, Na, and K were 14.6 ± 8.3 (± 1*σ* SD), 3.4 ± 3.1, 10.6 ± 10.6, and 1.7 ± 1.6 mg/L, respectively. The order from the highest content was Ca > Na > Mg > K, and the dominant cation was mainly Ca^2+^. For major anions, the average concentrations of Cl^−^, NO_3_^−^, and SO_4_^2−^ were 13.6 ± 24.4, 3.6 ± 4.7, and 13.9 ± 13.4 mg/L, respectively. As shown in Fig. 3, the highest concentration of all major components in Japan was found to be in Chiba Prefecture (for the location of the prefecture, see Fig. S1). Compared with the collected tap water in 33 other countries, the Japanese tap water concentration values were found to have low major components (Fig. 2). Furthermore, comparing the concentrations of major components in Japanese tap water with other published data^3,4,7^, we found that the Japanese tap water showed rather low average concentrations. These differences may have been caused by the raw water quality, geographic and seasonal conditions, and water treatment systems.

In Japan, tap water sources are mainly dam and river water^10^, whereas most nations’ drinking tap water is derived from groundwater^3,16–18^. Water is pumped up from the river at the sluice gate and gets purified at a water purification plant. The water is then delivered to each house through water pipes. The natural source of surface water is mainly rainwater. Rainwater dissolves into rivers or reacts with soil or rocks on the ground and dissolves into groundwater and flows into rivers. In Japan, approximately 70% of the country consists of mountains with a steep topography^19,20^. Consequently, rainfall runs off rapidly. Furthermore, Japanese rivers are shorter and steeper than those in other countries^21^; thus, the water flows rapidly down the mountains, across plains, and into the ocean, resulting in fewer water components. This is the reason for the difference in tap water quality between Japan and other countries.

### Water hardness in Japan

Water hardness is caused by various dissolved metallic ions, predominantly Ca and Mg ions. In other words, hard water has high amounts of dissolved minerals, especially Ca- and Mg-bearing minerals^22–24^. The water hardness is calculated from analytical data using Eq. (1)^23,25^:

1 $${\text{Total-hardness }}\left( {{\text{mg}}/{\text{L as CaCO}}_{{3}} } \right) \, = {\text{ Ca }}\left( {{\text{mg}}/{\text{L}}} \right) \, \times { 2}.{497 } + {\text{ Mg }}\left( {{\text{mg}}/{\text{L}}} \right) \, \times { 4}.{118}$$

Water hardness is an important and highly relevant issue for consumers because of its impact on taste^22,26^. Furthermore, the differences in water hardness affect not only taste but also soap-foaming and scaling in pipelines.

Boxplot of tap water hardness in Japan is shown in Fig. 3. The distribution of tap water hardness in Japan varied widely, ranging from 0.1 to 511 mg/L, with an average of 50.5 ± 30.2 mg/L (*n* = 1564). Compared with other countries, Japanese tap water hardness exhibited soft water with low major component concentrations (Fig. 2). Only one point exceeded the Japanese standard value (300 mg/L) in Chiba, with a concentration of 511 mg/L, which was set not for health risks but rather for the prevention of scale generation in pipelines.

The water hardness in Japanese tap water tended to be higher in the Kanto region, particularly in Chiba and Saitama (see Fig. S1 for the prefecture and region). Chiba Prefecture exhibited the highest hardness, with an average of 97.4 ± 57.2 mg/L (*n* = 75). Moreover, the major components had the highest concentrations in Chiba as well, as shown in Fig. 3. The main water source for these prefectures relies on the Tone River system (Tone River and Edo River). The Tone River basin features a high population, and rivers flow into human activity areas^27,28^. Moreover, these prefectures are located downstream of the Tone River and exhibit a long residence time before being taken in as raw water. Anthropogenic activities, such as the intensification of urban and industrial sectors and agricultural practices, affect hydrochemical properties by enhancing cation and anion properties and heavy metal contamination^29–31^. Consequently, various component inputs (e.g., wastewater, industrial effluents, and agricultural water) flow into rivers, resulting in higher dissolved components and thus increased water hardness. Furthermore, the geological factor contributes to the high water hardness in the Kanto region. The limestone geology in the upstream area of the Tone River also contributes to the high water hardness. Additionally, the major aquifers of the Kanto plain comprise quaternary sediments and, in a geological context, represent a new environment where Ca and Mg are easily eluted^20,32^. Groundwater has a long residence time because of the large plains in the Kanto region. For these reasons, the tap water in the Kanto region is considered to be relatively hard.

Meanwhile, Kumamoto’s tap water exhibited a generally high hardness, with an average of 66.3 ± 28.4 mg/L, as shown in Fig. 3. In Kumamoto, the source of tap water is mainly groundwater (79%), which passes through the permeable pyroclastic flow deposits of Mount Aso (Aso-1, 2, 3, and 4) and springs rich in ionic species^32,33^. Tap water in Okinawa also has high hardness, with an average of 59.4 ± 35.3 mg/L, because it is derived from surface water and groundwater passes through Ryukyu’s limestone geology^32^.

In comparison, in the northern side of Japan, snowfall regions tended to have low hardness because the raw water contained snowmelt water. The tap water hardness was widely distributed throughout Japan, although Japan’s water is generally regarded as soft according to WHO classification (Fig. 3)^24^. Thus, some consumers may notice a change in the taste of tap water due to the wide domestic distribution of hardness.

### Japanese tap water quality

All tap water in Japan must meet regulations for the concentration of 51 components related to water safety; that is, not even one parameter must exceed the regulation value^36^. In this study, most of the analyzed tap water did not exceed the drinking water quality standards based on the Japanese waterworks law and WHO guidelines, as shown in Table 1^22,34–36^. Approximately 1% (17/1564 sites) of the samples in this study exceeded the Japanese standard values. No exceedances of standard items were detected for Al (0.2 mg/L), Cu (1.0 mg/L), and Cr (0.02 mg/L as Cr(VI)).

Fe was detected in 36% of all the analyzed samples (*n* = 1564), and the number exceeding the Japanese standard value (0.3 mg/L) was only one site, with a concentration of 0.43 mg/L. Meanwhile, Zn was detected in 85% of the samples, with eight sites exceeding the Japanese standard (1.0 mg/L) and one site exceeding the WHO guidelines (3 mg/L). Pb was detected in 0.3% (five sites) of the samples, and all five sites exceeded the Japanese standard value and WHO guideline value (0.01 mg/L). In three of the five sites where Pb exceeded the standard value, Zn also exceeded the standard value, with concentrations of 1.28, 2.48, and 1.62 mg/L, respectively. Mn exceeded the standard value (0.05 mg/L) at four sites, and Cl^−^ exceeded the standard value (200 mg/L) at the same four sites. It was suggested that seawater or deep well water was the source of tap water at these four sites. In addition, a site with both Mn and Cl^−^ exceedances had exceedances of Na and hardness. Because this site was located in a coastal area, seawater was considered to have flowed into the raw water. For NO_3_^−^, exceedances to the Japanese standard (10 mg/L as NO_3_–N) and WHO guideline value (50 mg/L) were found at one site using a well water (groundwater) source. For Ni, the normative standard value was not set, but the guideline value (0.02 mg/L) was set as complementary items in Japanese law^35^. The guideline value was exceeded in 1.4% of the sites (21 sites), whereas the WHO guideline value (0.07 mg/L) was exceeded in four sites. At two of these 21 sites, Zn and Pb also exceeded the standard values. Cd was detected at one site, with a concentration that equaled the Japanese standard value of 0.003 mg/L. At this site, Zn was also detected with a high concentration of 1.44 mg/L.

Furthermore, some of the tap water we collected from other countries got turbid and developed suspended substances during storage in the laboratory for several weeks. These things did not occur at all with Japanese tap water, indicating satisfactory sanitary conditions.

Overall, the results of this study indicated that the quality of tap water in Japan is generally good. The sites where even one component exceeded the standard value were not concentrated in a specific water source and/or region. Furthermore, the faucets that exceeded the standard values were installed in various types of facilities, including private residences, accommodation facilities, public facilities, and restaurants. These results showed that the exceedances of water quality standards were not due to regional variations but were rather caused by specific influences within the supply process.

### Annual variation in water quality

To investigate the annual variations in water quality in major and trace components, tap water samples were collected from three households in Japan. Sampling was conducted from April 2021 to March 2023 (1.5 years for Musashino City), with water collected twice a month from the same tap. The selected sites were Niigata City (Niigata Prefecture), Suginami City (Tokyo), and Musashino City (Tokyo). The sources of tap water in Niigata City and Suginami City relied on surface water. Niigata City is located downstream of the Shinano River, the longest river in Japan, and uses its river water as raw water. Its upstream is an area with great snowfall. Meanwhile, tap water in Suginami City is derived from downstream of the Tone River system, which has a large basin population and is distributed through a municipal pipeline in the capital city of Tokyo. In contrast, the water supply in Musashino City relies primarily on groundwater as its main water source, with a mixture of 80% groundwater and 20% surface water.

**Figure 4:**
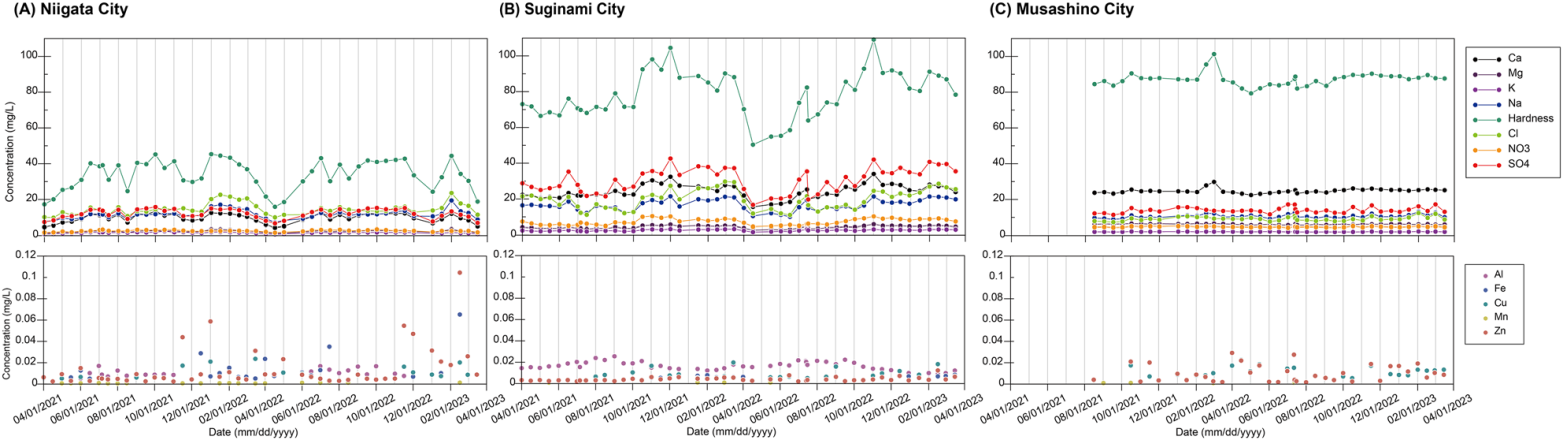
Annual concentration variations of inorganic components in household tap water during the observation period at (**A**) Niigata City, (**B**) Suginami City, Tokyo, and (**C**) Musashino City, Tokyo.

The water quality variations at the three sites are presented in Fig. 4 and Table S1. Throughout the study period, none of the locations exceeded the water quality standard value in Japan. The variations of major components, including Ca, K, Mg, Na, Cl^−^, NO_3_^−^, and SO_4_^2−^, exhibited similar patterns among the components, as shown in Fig. 4. The fluctuation of concentrations was assessed using the coefficient of variation (CV). The variation of major components tended to be higher in Niigata and Suginami, where the source water was surface water. For instance, the CVs for Ca (CV_Ca_) were 25% in Niigata, 16% in Suginami, and 5.1% in Musashino City. The water sourced from surface water showed approximately 20% concentration fluctuations for each component, whereas groundwater-derived tap water showed 5%–10% concentration fluctuations. The surface water sources for tap water exhibited larger annual concentration fluctuations compared with groundwater sources because of prolonged residence times of river water up to the point of intake due to the extensive flow path. In the case of river water as raw water, human activities in the surrounding areas, such as the inflow of domestic wastewater, industrial water, and agricultural water, affect water quality^29–31,37,38^. In addition, the concentrations of major components were higher in Suginami than in Niigata. This discrepancy was attributed to the higher population density in the Tone River basin, which serves as the water source for Suginami, resulting in increased human activities and associated influences. Moreover, in areas with surface water sources, the concentrations tended to decrease in spring because of dilution effects from snowmelt in the upstream regions. In contrast, groundwater quality was influenced by geology and less by human activities. Tap water derived from groundwater is considered to be less influenced by human activities, resulting in minimal annual variations of water quality, such as that in Musashino City. Thus, major components in tap water from surface water sources would have greater annual concentration variations than those from groundwater sources.

Trace metal elements, including Al, Fe, Cu, Mn, and Zn, were not detected continuously during the sampling period. The behavior of annual variations differed from that of the major components. The CVs for Cu (Cv_Cu_) were 48% in Niigata, 46% in Suginami, and 36% in Musashino, whereas those for Zn (CV_Zn_) were 134% in Niigata, 45% in Suginami, and 78% in Musashino. The fluctuations in trace elements were larger than those in major components. Moreover, the annual variations among the major components showed a good correlation (Table S2). However, the fluctuations in trace elements showed a different pattern from those in major components. In this case, the variation of trace elements showed no clear correlation between the major components and trace elements in water quality fluctuations.

To evaluate the water quality on the same day and within the same supply area, tap water samples were collected from two different points in Ota Ward, Tokyo. The concentration results are shown in Table 2. At Point 1, the concentrations of Zn and Cu were 0.022 and 0.025 mg/L, respectively. However, at Point 2, the concentration of Zn was 0.003 mg/L, whereas Cu was not detected, with a detection limit of 0.005 mg/L. Although the samples were collected in the same supply area on the same day, the concentration of Zn was approximately 7.3 times the difference between the two points. In contrast, no significant differences were found in the concentrations of major components, such as Ca and Mg, suggesting that factors influencing trace element behavior differed from those affecting major components.

**Table 2:** Concentrations of major and trace components in the same water supply area (unit: mg/L).

	Ca	K	Mg	Na	Cl^−^	NO_3_^−^	SO_4_^2−^	Br^−^	Sr	Al	Zn	Cu	Fe	Mn
Point 1	23.0	3.0	4.6	13.0	14.7	8.1	29.5	0.05	0.073	0.012	0.022	0.025	n.d.	n.d.
Point 2	24.4	3.1	5.0	14.9	16.6	9.1	33.3	0.06	0.078	0.012	0.003	n.d.	n.d.	n.d.

We reported that the tap water quality of major components (e.g., Ca, Mg, and hardness) is derived from that of raw water^15^. These results indicated that the behavior of trace elements is not dependent on raw water quality or regional differences because raw water quality tends to be exhibited in tap water. Although the source of trace metal elements is natural waters, additional sources are plumbing pipes in most cases. For this reason, the detection of trace elements is considered to be influenced by localized factors, such as supply pipes. Concentration fluctuations of major components in tap water are derived from fluctuations in raw water. However, trace elements suggested by the plumbing system as the main source are influenced by local infrastructure factors, such as supply pipes near the tap, rather than raw water quality or regional variations.

In summary, tap water derived from surface water sources exhibited larger annual concentration fluctuations, and major components showed varying concentrations influenced by the natural environment and human activities. The results for major components, such as Ca and Mg, reflected the quality of the water sources. Trace elements were more affected by local infrastructure, indicating that their fluctuations were not solely tied to raw water quality or regional variations.

### Characteristics of Japanese tap water

The Piper trilinear diagram was used to categorize Japanese tap water types according to the contents of major cations (Ca^+^, Mg^2+^, K^+^, and Na^+^) and major anions (Cl^−^, SO_4_^2−^, and HCO_3_^−^)^39^. The Piper diagram consists of a central key diamond diagram and two triangular diagrams, showing the relative abundance of major ions. The left triangle shows the relative abundance of cations, whereas anion facies are shown on the right triangle. The central diamond diagram exhibits the water type, which projects binary plots of cation and anion facies. All major ion concentrations were converted to meq/L to construct the Piper diagrams. In this study, we could not analyze HCO_3_^−^ directly because of our equipment. Thus, HCO_3_^−^ concentration was calculated as follows (Eq. 2), without considering the effect of organic acid anions^40^:

2 $${\text{HCO}}_{{3}}{^{ - }} = {\text{ cation sum }}\left( {{\text{Ca}}^{ + } + {\text{ Mg}}^{{{2} + }} + {\text{ K}}^{ + } + {\text{ Na}}^{ + } } \right) - {\text{anion sum }}({\text{Cl}}^{ - } + {\text{ NO}}_{{3}}^{ - } + {\text{ SO}}_{{4}}{^{{2 - }}} ){\text{ meq}}/{\text{L}}$$

**Figure 5:**
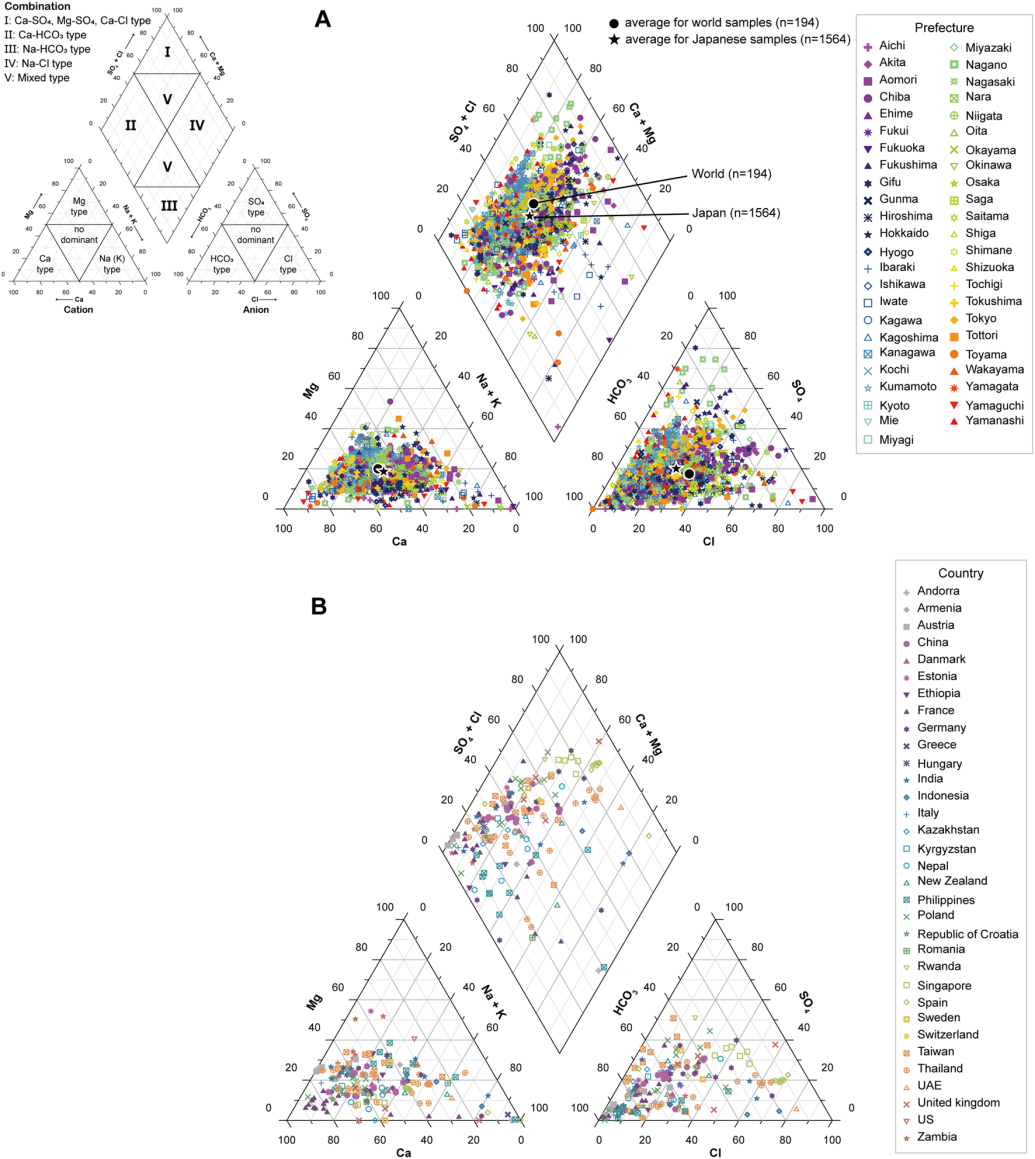
Piper trilinear diagram showing the relative abundances of major ions and water type characteristics. (**A**) Japanese tap water (*n* = 1564) by prefecture indicated together with the average for tap water samples collected in Japan (filled star) and the world (filled circle); (**B**) worldwide tap water collected from 33 countries (*n* = 194).

A Piper trilinear diagram for all 1564 analyzed Japanese tap water samples is shown in Fig. 5A. In the Japanese water samples, the majority of cation facies decreased in the following order: Ca > no dominant > Na + K > Mg. The dominant cation showed Ca composition in 50% of the tap water samples, whereas there was no dominant type in 43% of the samples. The majority of anion facies decreased in the following order: HCO_3_ > no dominant > Cl > SO_4_. The dominant anions were mainly HCO_3_ in 61% of the samples, whereas 32% of the samples had no dominant type. As shown in the diamond part, most of the Japanese tap water fell in the combination zones II and V, indicating that the majority of tap water was of Ca–HCO_3_ type and mixed type with Ca–SO_4_ and Ca–Cl types based on the Ca–HCO_3_ type. Water with high Ca had dominant bicarbonate. In the case of the outlier type, we assumed the installation of other water purification systems. The Ca–HCO_3_ type mainly originated from shallow groundwater. Meanwhile, surface water was mainly used for tap water supply in Japan, with well water being used or mixed as a water source through the supply process. Consequently, the types of Japanese tap water showed a Ca–HCO_3_ type or a mixed type.

Figure 5B shows the diagram of tap water samples collected from 33 other countries except Japan. These waters were similar to the Japanese tap water type based on the relative abundance of major ions. However, most of these tap waters had a dominant Ca–HCO_3_ type and higher Ca and bicarbonate compositions than the Japanese tap water. Furthermore, the mixed type and no dominant facies of major ions occurred less often than in Japanese tap water because most nations’ tap water was derived from groundwater^2,3^. Generally, many surface waters and most shallow groundwaters are dominated by the Ca–(Mg)–HCO_3_ type because of the relatively rapid kinetic weathering of carbonate minerals^4^. Especially in Europe, tap water supplied from limestone geologies exhibits a high Ca content and thus becomes water with high Ca relative abundance^3^. Additionally, the majority of potable or drinking water is of the Ca–HCO_3_ type, which is more suitable for drinking purposes. Therefore, Japanese tap water was of the same type as those in other countries and was suitable for drinking.

## Conclusion

From 2019 to 2024, drinking tap water samples were collected from 1564 sites across Japan, with an additional 194 samples collected from 33 other countries, to conduct a comprehensive survey of tap water quality in Japan. Tap water samples were analyzed for 27 inorganic components, with a primary focus on the distribution of major components (Ca, Mg, Na, K, Cl^−^, NO_3_^−^, and SO_4_^2−^) and trace metal elements (Al, Fe, Cu, Mn, and Zn). Although most components showed wide concentration ranges, Japanese tap water had fewer major components compared with those from other countries. The detection trends of trace elements varied across supply faucets. Long-term monitoring of tap water quality at the same faucets revealed fluctuations in trace metal concentrations distinct from those of the major components. This suggests that the behavior of trace elements is influenced by local infrastructure, such as supply pipes, distinct from the variability of raw water quality.

The use of the Piper diagram to evaluate water types revealed that Japanese tap water is predominantly categorized by Ca–HCO_3_ and mixed types. Japanese tap water generally complies with water quality standards, with only 1% (17/1564 sites) exceeding the standard criteria, which were scattered without regional bias. These results indicated the provision of reliable and high-quality tap water for the general public in Japan.

## Methods

### Sample collection

**Figure 6:**
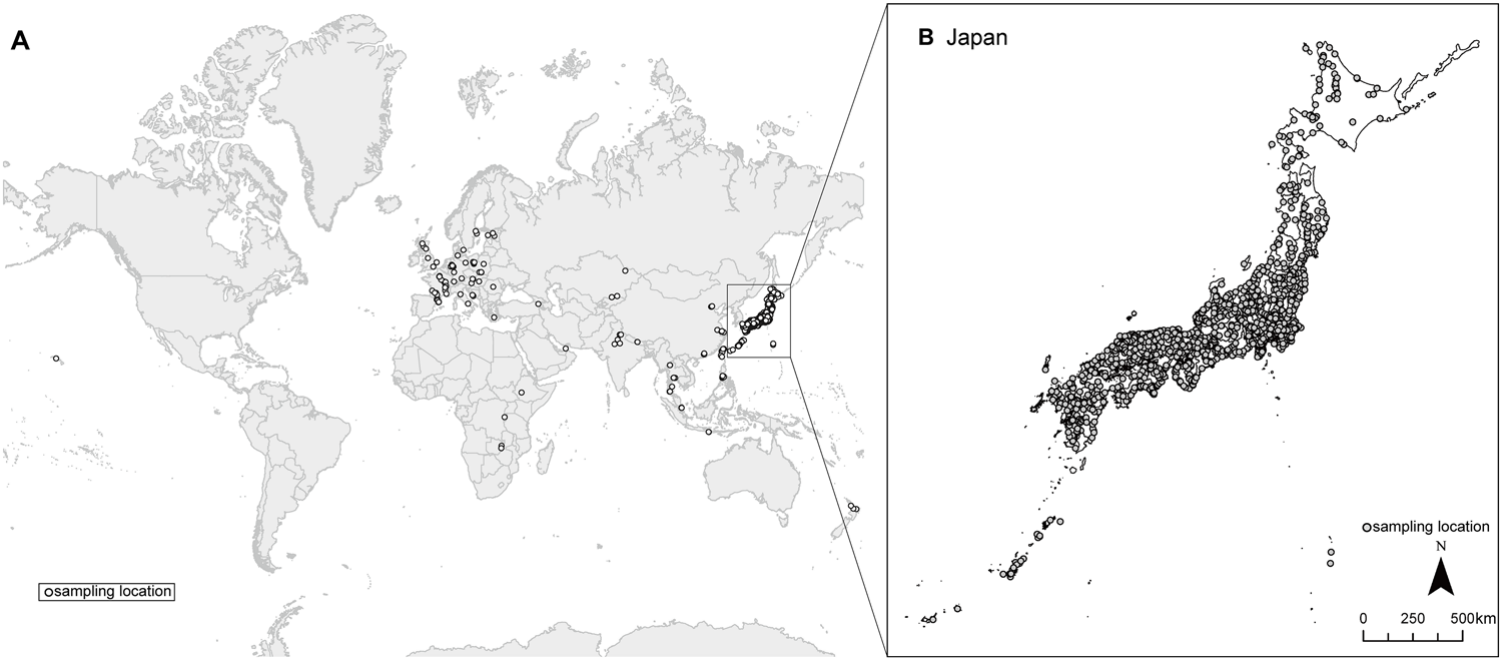
Map of the locations of (**A**) all sampling sites in 34 countries (*n* = 1758) and (**B**) sampling sites in Japan (*n* = 1564). The maps were generated using ArcGIS Pro, version 2.9.3 (ESRI).

A total of 1564 tap water samples were collected from all 47 prefectures of Japan from 2019 to 2024. Furthermore, we also collected samples from 194 sites in 33 other countries during the study period. The locations of the sources of the tap water samples are shown in Fig. 6. To include many different areas, sampling was conducted not only by our research team but also by other researchers, colleagues, and friends who supported the importance of this study.

Tap water samples were taken from private homes and public spaces (offices, restaurants, public facilities, train stations, airports, or highway service stations) supplied via a public (municipal) plumbing system throughout Japan without distinguishing private/public facilities. When collecting tap water, details about the condition sources were not specified; this was only performed for samples taken from water pipes.

The tap water samples were collected directly from ordinary taps after running for several seconds (to avoid collecting stagnant water in the distribution system). The sampling strategy was directed at end-consumers, and we tried to collect the samples in the consumers’ normal drinking usage. Before sampling, polyethylene sampling bottles were rinsed three times with tap water and stored as filled bottles to prevent air bubbles as much as possible. Whenever possible, the water pH was measured in the field using a Merck MQuant StripeScan. No filtration was performed during sampling. All collected samples were transferred to the laboratory as soon as possible and stored at 4 °C.

### Analytical measurement

The analytical methodology referred to the Guidelines for Drinking Water Quality—Annex 4 set by the WHO^22^, and the analytical procedures for the water samples were performed based on the methods proposed by the American Public Health Association (APHA)^25^. The collected water samples were analyzed for 22 elements using ICP-OES (Agilent, ICP-OES 720): Al, Ba, Be, Bi, Ca, Cd, Co, Cr, Cu, Fe, Ga, K, Li, Mg, Mn, Na, Ni, Pb, Se, Sr, Tl, and Zn. The concentrations of anion species (Br^−^, Cl^−^, NO_3_^−^, SO_4_^2−^, and PO_4_^3−^) were determined using IC (Shimadzu, Prominence). The standard solutions for calibration were prepared with multi-element standard solutions (Merck ICP Multi-Element Standard Solution VIII, Thermo Scientific Dionex Combined Seven Anion Standard II), and were appropriately diluted with ultrapure water (Millipore, Milli-Q, 18MΩcm). For quality control, elemental concentrations determined in the certified reference/standard solutions: SPEX CertiPrep XSTC-622 (35 multi-element standard) for ICP-OES, and Thermo Scientific™ Dionex™ Combined Seven Anion Standard I for IC were used to evaluate the uncertainty and recovery rates of the analysis. Target elements analyzed using ICP-OES were described as metals, such as Ca (not Ca^2+^), in several references^2–7^. Total-hardness was calculated from the analytical data.

### Statistical analysis

Exploratory data analysis was used to investigate the distribution of the dataset. Data from all sites were used in the analysis, and no points were intentionally excluded. Stats of the obtained results are presented as averages, standard deviations, medians, and ranges of concentrations, and figures are partly calculated/made using SPSS Statistics (ver. 17.0) and Microsoft Excel2019.

### Supplementary Information

Supplementary Information.

## Data Availability

The datasets analyzed during the current study are available from the corresponding author Dr. Mayumi Hori on reasonable request.
