# Supplementary material for: Distribution of inorganic compositions of Japanese tap water: a nationwide survey in 2019–2024
Source: Sci Rep. 2024 Jun 19;14:14167. doi: 10.1038/s41598-024-65013-4 (PMC11187173; doi:10.1038/s41598-024-65013-4)
Supplement: Supplementary file 1 — Supplementary Information. [file 41598_2024_65013_MOESM1_ESM.docx]

**Supplementary Information**

**Distribution of inorganic compositions of Japanese tap water: A nationwide survey in 2019–2024.**

Mayumi Hori^1*^, Katsumi Shozugawa^2^, Tsutomu Takizawa^2^, and Yuichiro Watanabe^1,2^

^1^ Komaba Organization for Educational Excellence, The University of Tokyo, 3-8-1 Komaba, Meguro, Tokyo, 153-8902, Japan

^2^ Graduate School of Arts and Sciences, The University of Tokyo, 3-8-1 Komaba, Meguro, Tokyo, 153-8902, Japan

*Corresponding author

Mayumi Hori

cmayumi@mail.ecc.u-tokyo.ac.jp

Contents:

Pages S1 to S4.

Figure S1. Map showing the names of prefectural regions in Japan. Parentheses following the prefecture name indicate the number of collected samples. The map was created using Adobe Illustrator 2024.

Table S1. Concentration results of inorganic components in three household tap water samples.

Table S2. Correlation coefficients for inorganic components. Top: Niigata City; middle: Suginami City; bottom: Musashino City


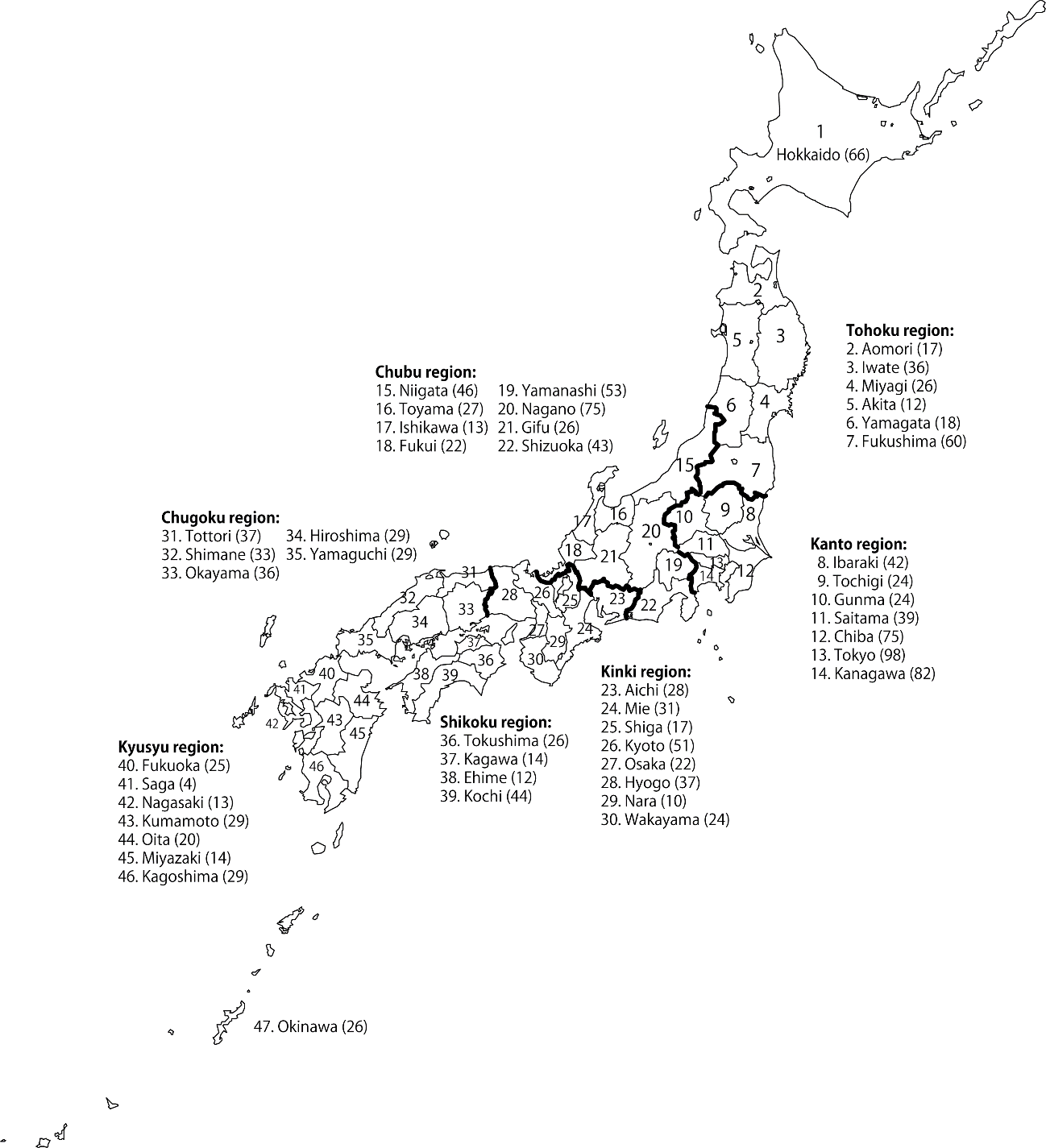


Figure S1. Map showing the names of prefectural regions in Japan. Parentheses following the prefecture name indicate the number of collected samples. The map was created using Adobe Illustrator 2024.

Table S1 Concentrations of inorganic components in three household tap water samples.

| Components | **Niigata City, Niigata** | | | **Suginami City, Tokyo** | | | **Musashino City, Tokyo** | | |
| --- | --- | --- | --- | --- | --- | --- | --- | --- | --- |
|  | Average (mg/L) | Range (Min–Max) | CV (%) | Average (mg/L) | Range (Min–Max) | CV (%) | Average (mg/L) | Range (Min–Max) | CV (%) |
| Ca | 9.7 | 4.2–13.4 | 25.0 | 24.4 | 15.6–34.0 | 15.6 | 24.8 | 22.5–29.8 | 5.1 |
| K | 1.6 | 0.7–2.5 | 22.4 | 2.7 | 1.7–3.4 | 15.5 | 2.1 | 2.0–2.3 | 4.0 |
| Mg | 2.4 | 1.3–3.5 | 22.7 | 4.3 | 2.8–5.9 | 17.2 | 6.1 | 5.6–6.5 | 3.9 |
| Na | 11.6 | 7.1–19.4 | 20.8 | 16.7 | 10.0–21.5 | 18.7 | 10.6 | 8.9–13.1 | 9.2 |
| Hardness | 34.1 | 15.9–45.3 | 24.0 | 78.7 | 50.4–109.1 | 15.9 | 87.1 | 79.3–101.3 | 4.2 |
| Cl- | 14.6 | 9.8–23.6 | 21.9 | 19.7 | 11.1–29.7 | 26.9 | 9.1 | 7.3–12.4 | 12.5 |
| NO_3_^-^ | 2.7 | 1.6–3.5 | 17.0 | 7.3 | 4.6–10.4 | 22.7 | 4.8 | 4.3–5.3 | 5.2 |
| SO_4_^2-^ | 12.2 | 6.6–15.8 | 21.0 | 30.7 | 16.7–42.6 | 21.6 | 14 | 11.4–17.2 | 10.8 |
| Al | 0.010 | n.d.(<0.005)–0.017 | 31.7 | 0.016 | 0.008–0.026 | 24.6 | n.d. (< 0.005) | — | — |
| Fe | 0.013 | n.d.(<0.005)–0.065 | 94.5 | 0.006 | n.d. (<0.005)–0.008 | 12.6 | n.d. (< 0.005) | — | — |
| Cu | 0.012 | n.d.(<0.005)–0.023 | 47.9 | 0.009 | n.d.(<0.005)–0.020 | 46.2 | 0.012 | n.d.(<0.005)–0.023 | 36.3 |
| Mn | 0.001 | n.d.(<0.001)–0.001 | 30.3 | 0.001 | n.d.(<0.001)–0.001 | 4.8 | 0.001 | n.d.(<0.001)–0.002 | 32.0 |
| Zn | 0.014 | 0.002–0.104 | 134 | 0.004 | n.d.(<0.001)–0.012 | 45.0 | 0.010 | n.d.(<0.001)–0.029 | 78.0 |

Table S2 Correlation coefficients for inorganic components. Top: Niigata City; middle: Suginami City; bottom: Musashino City

| **Niigata City** | Ca | K | Mg | Na | Hardness | Cl^−^ | NO_3_^−^ | SO_4_^2−^ | Al | Fe | Cu | Mn | Zn |
| --- | --- | --- | --- | --- | --- | --- | --- | --- | --- | --- | --- | --- | --- |
| Ca |  |  |  |  |  |  |  |  |  |  |  |  |  |
| K | 0.88 |  |  |  |  |  |  |  |  |  |  |  |  |
| Mg | 0.93 | 0.78 |  |  |  |  |  |  |  |  |  |  |  |
| Na | 0.74 | 0.64 | 0.92 |  |  |  |  |  |  |  |  |  |  |
| Hardness | 0.99 | 0.86 | 0.96 | 0.80 |  |  |  |  |  |  |  |  |  |
| Cl^−^ | 0.59 | 0.52 | 0.80 | 0.96 | 0.66 |  |  |  |  |  |  |  |  |
| NO_3_^−^ | 0.82 | 0.83 | 0.66 | 0.44 | 0.79 | 0.28 |  |  |  |  |  |  |  |
| SO_4_^2−^ | 0.98 | 0.85 | 0.88 | 0.67 | 0.97 | 0.51 | 0.82 |  |  |  |  |  |  |
| Al | 0.19 | 0.16 | 0.13 | 0.10 | 0.17 | 0.11 | 0.34 | 0.21 |  |  |  |  |  |
| Fe | −0.06 | −0.10 | −0.03 | 0.06 | −0.05 | 0.06 | 0.05 | −0.11 | 0.31 |  |  |  |  |
| Cu | 0.30 | 0.33 | 0.46 | 0.55 | 0.35 | 0.70 | 0.08 | 0.22 | 0.08 | 0.34 |  |  |  |
| Mn | 0.17 | 0.15 | 0.09 | 0.07 | 0.15 | 0.02 | 0.41 | 0.15 | 0.48 | 0.56 | 0.01 |  |  |
| Zn | −0.02 | −0.05 | 0.13 | 0.21 | 0.02 | 0.26 | −0.12 | −0.07 | −0.36 | 0.58 | 0.71 | 0.18 |  |
|  |  |  |  |  |  |  |  |  |  |  |  |  |  |
| **Suginami City** | Ca | K | Mg | Na | Hardness | Cl^−^ | NO_3_^−^ | SO_4_^2−^ | Al | Fe | Cu | Mn | Zn |
| Ca |  |  |  |  |  |  |  |  |  |  |  |  |  |
| K | 0.82 |  |  |  |  |  |  |  |  |  |  |  |  |
| Mg | 0.96 | 0.89 |  |  |  |  |  |  |  |  |  |  |  |
| Na | 0.78 | 0.92 | 0.88 |  |  |  |  |  |  |  |  |  |  |
| Hardness | 1.00 | 0.85 | 0.98 | 0.81 |  |  |  |  |  |  |  |  |  |
| Cl^−^ | 0.67 | 0.85 | 0.81 | 0.95 | 0.71 |  |  |  |  |  |  |  |  |
| NO_3_^−^ | 0.88 | 0.73 | 0.88 | 0.66 | 0.89 | 0.63 |  |  |  |  |  |  |  |
| SO_4_^2−^ | 0.88 | 0.88 | 0.94 | 0.90 | 0.90 | 0.85 | 0.75 |  |  |  |  |  |  |
| Al | −0.30 | −0.35 | −0.44 | −0.47 | −0.33 | −0.61 | −0.50 | −0.47 |  |  |  |  |  |
| Fe | −0.24 | 0.02 | −0.07 | 0.14 | −0.20 | 0.19 | −0.20 | 0.12 | −0.49 |  |  |  |  |
| Cu | 0.32 | 0.45 | 0.36 | 0.32 | 0.33 | 0.34 | 0.39 | 0.32 | −0.19 | −0.22 |  |  |  |
| Mn | 0.40 | 0.40 | 0.49 | 0.31 | 0.43 | 0.23 | 0.50 | 0.51 | −0.68 | 1.00 | −0.88 |  |  |
| Zn | 0.23 | 0.33 | 0.31 | 0.29 | 0.25 | 0.38 | 0.37 | 0.31 | −0.50 | 0.03 | 0.59 | 0.33 |  |
|  |  |  |  |  |  |  |  |  |  |  |  |  |  |
| **Musashino City** | Ca | K | Mg | Na | Hardness | Cl^−^ | NO_3_^−^ | SO_4_^2−^ | Al | Fe | Cu | Mn | Zn |
| Ca |  |  |  |  |  |  |  |  |  |  |  |  |  |
| K | 0.22 |  |  |  |  |  |  |  |  |  |  |  |  |
| Mg | 0.43 | 0.33 |  |  |  |  |  |  |  |  |  |  |  |
| Na | 0.52 | 0.55 | −0.11 |  |  |  |  |  |  |  |  |  |  |
| Hardness | 0.97 | 0.27 | 0.63 | 0.42 |  |  |  |  |  |  |  |  |  |
| Cl^−^ | 0.29 | 0.64 | −0.04 | 0.91 | 0.24 |  |  |  |  |  |  |  |  |
| NO_3_^−^ | 0.47 | 0.61 | 0.34 | 0.54 | 0.49 | 0.60 |  |  |  |  |  |  |  |
| SO_4_^2−^ | 0.26 | 0.50 | −0.21 | 0.83 | 0.16 | 0.85 | 0.56 |  |  |  |  |  |  |
| Al | — | — | — | — | — | — | — | — |  |  |  |  |  |
| Fe | — | — | — | — | — | — | — | — | — |  |  |  |  |
| Cu | −0.36 | 0.26 | −0.31 | 0.12 | −0.37 | 0.12 | −0.20 | 0.12 | — | — |  |  |  |
| Mn | −0.27 | −0.23 | −0.30 | −0.02 | −0.29 | 0.24 | −0.14 | 0.19 | — | — | 0.78 |  |  |
| Zn | −0.08 | 0.26 | 0.00 | 0.33 | −0.07 | 0.39 | 0.10 | 0.32 | — | — | 0.60 | −0.36 |  |
